# Effects of Trap Color and Placement Height on the Capture of Ambrosia Beetles in Pecan Orchards

**DOI:** 10.3390/insects16060569

**Published:** 2025-05-28

**Authors:** Rajendra Acharya, Shivakumar Veerlapati, Madhav Koirala, Andrew Sawyer, Apurba K. Barman

**Affiliations:** 1 Department of Entomology, University of Georgia, Tifton, GA 31793, USA; racharya@uga.edu (R.A.); shivakumar.veerlapati@uga.edu (S.V.); mkoirala@uga.edu (M.K.); 2 University of Georgia Extension, Statesboro, GA 30458, USA; agsawyer@uga.edu

**Keywords:** ambrosia beetles, *Xylosandrus crassiusculus*, trap color, trap height, monitoring, pecan

## Abstract

Ambrosia beetles (Coleoptera: Curculionidae: Scolytinae) in the tribe Xyleborini are economically important pests of woody ornamentals, tree nuts, and fruit orchards, including pecans in the United States. Among them, the granulate ambrosia beetle, *Xylosandrus crassiusculus* (Motschulsky), is the most common species in pecan orchards in Georgia. Various traps, including ethanol-mediated Lindgren multi-funnel traps, panel traps, bottle traps, sticky cards, and ethanol-infused wooden bolts, are used in ambrosia beetle monitoring programs. Trap color and placement height are important factors that increase trap effectiveness. To improve trap effectiveness for ambrosia beetles, we conducted a color and height preference experiment under field conditions using six different colored sticky cards, including black, blue, green, red, transparent, and yellow, placing them at three different heights (15, 60, and 120 cm from ground level). The results show that red and transparent sticky cards consistently captured a higher number of ambrosia beetles, whereas yellow-colored sticky cards consistently captured a lower number of ambrosia beetles compared to all other tested colors of sticky cards. A similar trend was observed with *X. crassiusculus* in field and laboratory settings. Among the evaluated trap heights, more ambrosia beetles, including *X. crassiusculus*, were consistently captured in the sticky cards placed at a height of 60 cm from the ground surface. Additionally, we monitored natural infestations of ambrosia beetles in commercial pecan orchards in Georgia and found more damage to pecan trees near the ground surface (45 cm) compared to the upper parts. We also recorded three ambrosia beetle species, *X. crassiusculus*, the black stem borer, *X. germanus* (Blandford), and the Southeast Asian ambrosia beetle, *Xylosandrus amputatus* (Blandford). Among them, *X. crassiusculus* (90.50%) was the most abundant species in the pecan orchards. Therefore, red and transparent sticky cards placed at a height of 45 to 60 cm could improve the trap efficacy and can be used for monitoring ambrosia beetles in pecan orchards.

## Simple Summary

Ambrosia beetles are serious pests of various woody plants, including pecans. Monitoring their activity in orchards is crucial for making timely and effective management decisions. To optimize current monitoring efforts, this study evaluates the color and height preferences of ambrosia beetles in pecan orchards using colored sticky cards—specifically blue, black, green, red, yellow, and transparent—placed at three different heights: 15, 60, and 120 cm above ground level. The results show that red and transparent sticky cards placed at a height of 60 cm were highly effective in capturing ambrosia beetles. Our study provides valuable insights into the color and height preferences of ambrosia beetles that can be used to improve the monitoring and trapping efficiency of this pest in pecan and other crop production systems.

## 1. Introduction

Pecan [*Carya illinoinensis* (Wangenh.) K. Koch] is an important economic crop in the Unites States, with a value of U$ 460 million, contributing to approximately 80% of global supply [1,2]. However, pecan trees are susceptible to various insect pests, including ambrosia beetles. Fungus-farming ambrosia beetles (Coleoptera: Curculionidae: Scolytinae), in the tribe Xyleborini, are small woodborers and destructive pests, known to damage various economically important tree plant species worldwide, including pecans [3,4,5,6,7,8]. In the United States, two non-native ambrosia beetle species, the granulate ambrosia beetle, *Xylosandrus crassiusculus* (Motschulsky), and the black stem borer, *X. germanus* (Blandford), are common in tree nuts and fruit orchards, nurseries, and forests ecosystems [7,9,10,11,12]. Both species are native to South Asia and have a broad host range [9,13]. The overwintering adult female ambrosia beetles start searching for new host plants immediately after the daily temperature reaches 20–21 °C for a couple of days during early spring and infest new trees by tunneling into sapwood and heartwood [9,14,15]. They produce several galleries, inoculate their symbiotic fungi, oviposit on the fungal garden, and feed on fungal mycelia as both adults and developing larvae [16]. Affected trees display branch and canopy dieback, which can lead to tree death [17,18]. Many ambrosia beetle species, including *X. crassiusculus* and *X. germanus*, are attracted to the ethanol produced by stressed trees around the root zone in response to various biotic (pathogens) and abiotic stressors (freezing, flooding, and drought), which is used as a cue for host selection [4,19]. However, ethanol concentration greatly impacts the attack rate of these ambrosia beetle species [4].

Maintaining a healthy tree is key to preventing ambrosia beetle attacks in the orchards and nurseries [18]. It is challenging to manage ambrosia beetles once the trees are infested and have colonized the heartwood, even using systemic insecticides as the insects feed only on their symbiotic fungus [12]. Pyrethroid insecticides (bifenthrin, cypermethrin, and permethrin) are commonly applied to prevent attacks of ambrosia beetles in the field [18]. However, insecticide application timing can be challenging due to difficulty in detecting ambrosia beetles before they cause damage. Additionally, frequent preventative applications of insecticides might have non-target effects and may not be economical. Therefore, the development of effective monitoring techniques is needed to detect ambrosia beetles at low populations in the field so that the application of insecticides can be timely and effective. Several ethanol-baited commercial traps, including jar traps, Lindgren multi-funnel traps, panel traps, sticky traps, homemade bottle traps, and ethanol-infused wooden bolt traps, are available for monitoring ambrosia beetles [12,20,21]. Tobin et al. [21] reported that a clear sticky trap baited with an ethanol lure was the most effective for capturing ambrosia beetles compared to other traps, including bottle traps, jar traps, and panel traps. In addition to responding to host volatiles, such as ethanol, scolytine beetles, including ambrosia beetles, also use visual cues to locate their hosts [22,23]. Therefore, trap color may affect the capture of ambrosia beetles. Although some studies have been conducted to identify the color preference of ambrosia beetles, the results are inconsistent, and experiments were conducted using traps other than sticky cards, including bottle traps [24], panel traps [25], multiple-funnel traps [26], and prism traps [27].

Trap placement height is an important factor for monitoring ambrosia beetles [28,29,30]. In general, ambrosia beetle species are more abundant in the understory than the canopy [31,32]. However, attack height can differ among species of ambrosia beetles and trends for ambrosia beetle capture vs. trap height have been inconsistent [31,33,34,35]. Thus, further study is needed to understand the vertical distribution patterns of ambrosia beetles in the pecan production system.

In the present study, we evaluated the color and height preferences of ambrosia beetles using sticky traps to optimize the trapping protocol for efficient monitoring and to report the major ambrosia beetle species associated with natural infestations in the pecan production system in the state of Georgia.

## 2. Materials and Methods

### 2.1. Study Sites

The monitoring of natural ambrosia beetle infestations was performed in pecan orchards located near Chula (Irwin County; 31.648588, −83.488801), Quitman (Brooks County; 30.804520, −83.537667), Waycross (Ware County; 31.340081, −82.590156), and Portal (Bulloch County; 32.58385, −81.96854) in the state of Georgia during the growing season in 2023 and 2024.

The trap color and height preference experiments for ambrosia beetles were conducted in two commercial pecan orchards, Lenox (Latitude 31.310195 N, Longitude 83.488727 W) and Waycross (Latitude 31.340081 N, Longitude 82.590156 W), Georgia, between February and May 2024. The Lenox pecan orchard consisted of 8–10-year-old trees of Creek and Caddo cultivars, planted with 7.5 × 15 square meter (plant × row) spacing, whereas the Waycross pecan orchard consisted of 7–9-year-old trees of Lakota and Oconee cultivars, planted with 10.5 × 10.5 square meter (plant × row) spacing. In both locations, the woodlot adjacent to the pecan orchards consisted of a mix of hardwood and pine forest. There were no pesticide applications for ambrosia beetles during the experimental period.

### 2.2. Sticky Traps

Five different colors [black (RGB: 53, 52, 53), blue (RGB: 14.49, 149.76, 211.17), green (RGB: 95, 159, 84), red (RGB: 225, 71, 73), and yellow (RGB: 225, 213.11, 41.46)], as well as a transparent sticky card, were selected as the six “color” treatments for the experiment. For the purpose of this experiment, transparent cards were considered one of the treatment colors. Except for the transparent sticky cards, the other colored cards were prepared in the laboratory using colored paper (Astrobrights paper, Neenah Paper, Inc., Alpharetta, GA, USA). The transparent sticky cards were purchased from Trécé Inc., Adair, OK, USA. The paper was cut to 5.5 × 6 square inches and sealed with clear laminating film (Fellowes manufacturing company, Itasca, IL, USA). Afterwards, the cards were coated with transparent and odorless adhesive polymers (TAD-Trécé Adhesive Division, Adair, OK, USA) on both sides.

### 2.3. Monitoring of Natural Infestations of Ambrosia Beetles

Natural infestations of ambrosia beetles were monitored in four pecan orchards, and a total of 12 sample trees were used for this study (Table S1). Ambrosia beetle-infested pecan trees were cut near the soil surface and were brought to the lab. Information on the number of holes at each height and the ambrosia beetle species associated with the damage was recorded.

### 2.4. Field Experiment 1: Color Choice Experiment

**Figure 1:**
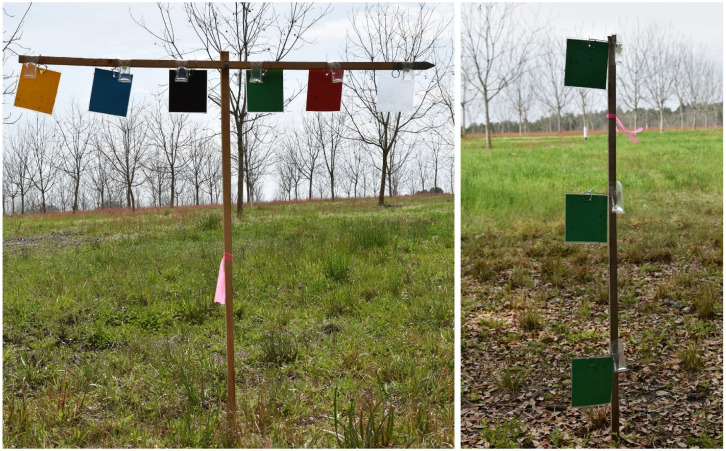
Experimental set-up for color (**left**) and height (**right**) preferences for ambrosia beetles in pecan orchards in Lenox and Waycross, Georgia.

A set of six colored sticky cards (black, blue, green, red, transparent, and yellow) were randomized vertically on the horizontal wooden pole using wire loops and were attached to the vertical wooden pole with the help of a screw (Figure 1), setting the height of the sticky cards at 15, 60, and 120 cm above ground level. The distance between each colored sticky card was 15 cm. Each set of six colored sticky cards at each trap height was replicated four times using a randomized complete block design.

In both experiments (the color and height choice experiments), each sticky card was baited with an AgBio low-release ethanol lure (16 mg/day at 20 °C; ChemTica International, Heredia, Costa Rica). The distance between each trap set within the block was 20 m, and traps were placed 10 m from the pecan orchard and oriented to face towards the woodlot. The sticky cards were replaced weekly over a period of 4 weeks. The sticky cards were collected individually in a clear poly bag (S-16831, Uline, Braselton, GA, USA), and Histo-Clear II (National Diagnostics, Atlanta, GA, USA) was utilized to remove the ambrosia beetles from the sticky cards for counting and species identification purposes. The ambrosia beetle species were identified using taxonomic keys [36,37,38].

### 2.5. Field Experiment 2: Height Choice Experiment

Each colored sticky card (black, blue, green, red, transparent, and yellow) was placed vertically on a wooden pole using wire loops at three different heights, 15, 60, and 120 cm from ground level (Figure 1), and was replicated four times using a randomized complete block design.

### 2.6. Lab Experiment: Color Choice Assay

The color choice experiment was conducted in an acari cage (92 × 61 × 61 cm) (l × b × h) under the following laboratory conditions: 23 ± 2 °C, 60 ± 5% RH, and 8:16 h light–dark. Six colored sticky cards were randomly placed on the cage floor without overlapping the cards, and each card was baited with AgBio low-release ethanol lures. Eighty adults of *X. crassiusculus* were released in the center of the cage in a 90 mm Petri dish, ensuring equal distance from each card. The number of *X. crassiusculus* attached to each sticky card was recorded after 24 h, and the experiment was repeated four times on different dates.

### 2.7. Statistical Analysis

The effects of trap color and trap height on the trap capture rate of ambrosia beetles were evaluated using a generalized linear mixed model (GLMM) with a negative binomial distribution utilizing the lme4 [39], car, and emmeans [40] packages available for R [41]. Data were analyzed according to the experimental site to determine the effects of trap colors and trap heights on the capture rate of total ambrosia beetles and *X. crassiusculus*. The trap colors and trap heights were designated as fixed effects and the block as a random effect in the model. The differences in total ambrosia beetle infestation rate (percentage attack rate) at various heights of the pecan trees from the natural infestation observations were evaluated using a GLMM (Tweedie family with a log link) utilizing the glmmTMB package because more values were zero in the dataset, especially at the upper height [42]. The tree height of pecan trees from ground level was treated as a fixed effect, whereas replications were treated as random effects. The laboratory-based color preference of *X. crassiusculus* data were analyzed using one-way ANOVA. Tukey’s HSD test (*p* < 0.05) was used to separate the mean values from the treatments.

## 3. Results

### 3.1. Monitoring of Natural Infestation of Ambrosia Beetles

**Figure 2:**
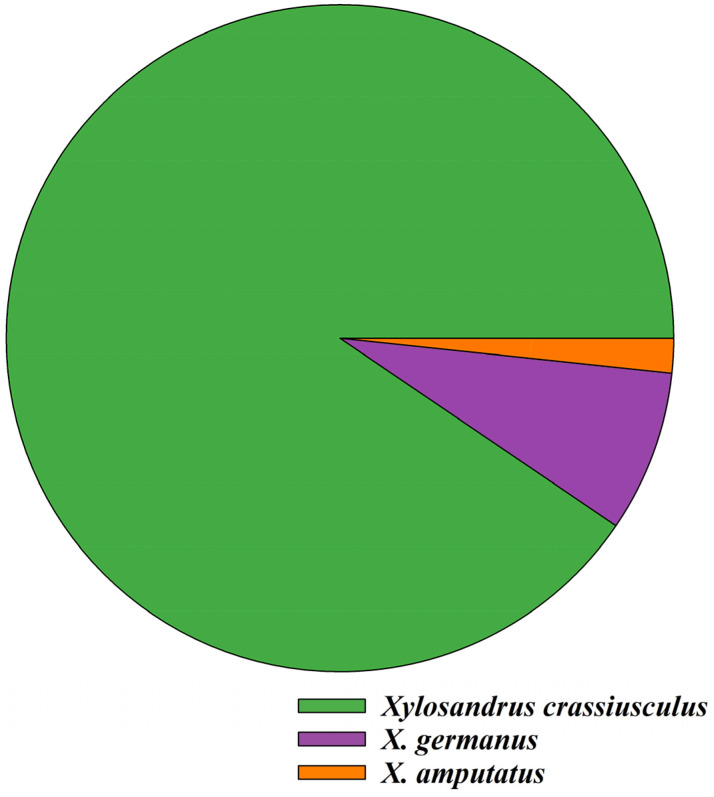
Percentage of ambrosia beetle species associated with natural infestations in pecan orchards in Georgia.

*Xylosandrus crassiusculus* (90.50%) was the dominant species, followed by *X. germanus* (7.83%) and *X. amputatus* (1.67%), in the pecan production system in Georgia (Figure 2, Table S1).

**Figure 3:**
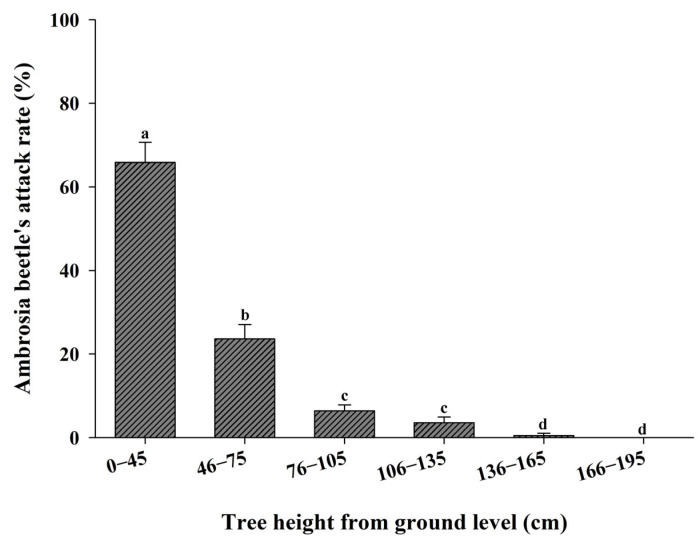
Percentage attacks (Mean ± SE) of ambrosia beetles in different heights of pecan trees from ground level in a natural infestation. Bars with different lower-case letters indicate significant differences in the ambrosia beetle’s attack rate among the infested parts of pecan trees (height) from the ground surface (*p* < 0.05, Tukey’s test).

The infestation of ambrosia beetles decreased with the height of pecan trees from the ground level. Significantly more attacks were observed in the lower portion of the tree (up to 45 cm from ground level) compared to the upper parts of the tree (*z* = 16.68; df = 5; *p* < 0.0001) (Figure 3). Ambrosia beetle entry holes were observed up to 165 cm from ground level. However, no entry hole was observed at 195 cm from ground level.

### 3.2. Ambrosia Beetle Diversity in the Pecan Orchards

**Figure 4:**
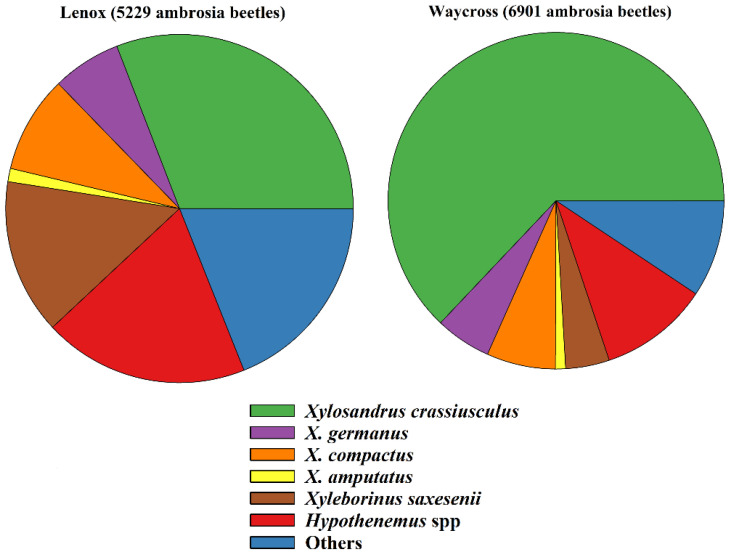
Percentage of ambrosia beetle species captured on sticky cards in pecan orchards in Lenox and Waycross, Georgia.

The total number of ambrosia beetles collected from both color and height choice experiments in Lenox and Waycross were 5229 and 6901, respectively. Of them, *X. crassiusculus* was the most abundant species in both locations, Lenox (30.89%) and Waycross (62.92%) (Figure 4, Table S2 and Table S3).

### 3.3. Color Preference of Ambrosia Beetles

**Figure 5:**
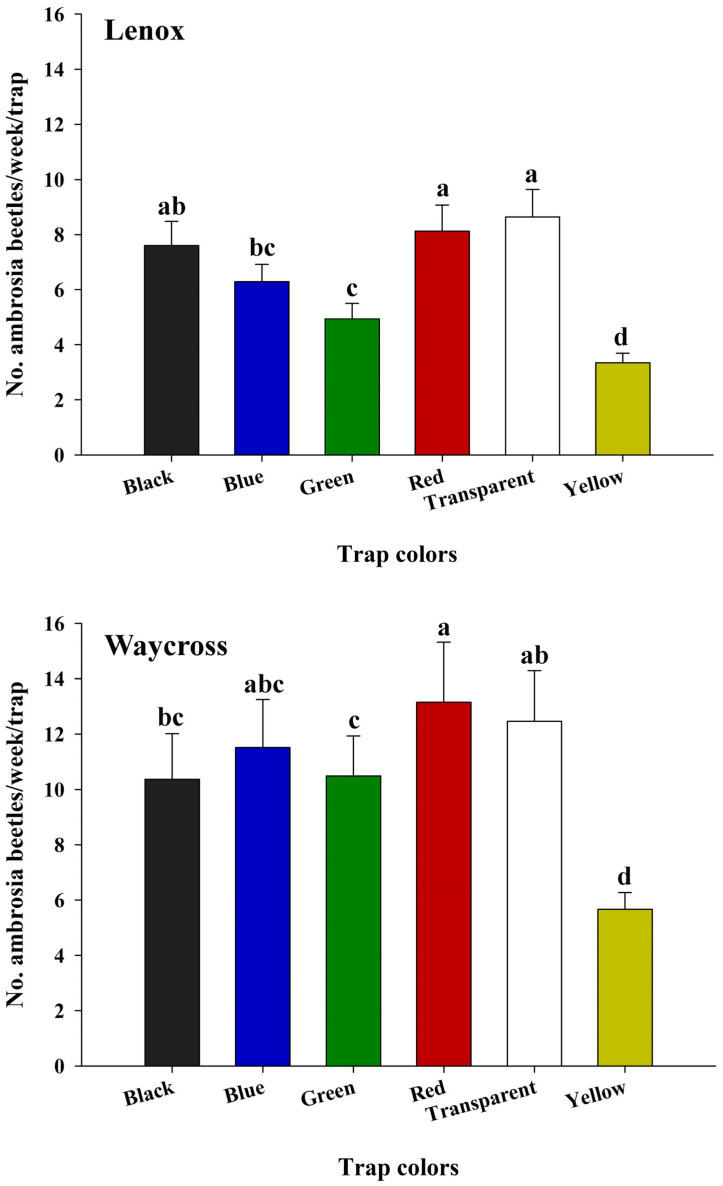
The number (mean ± SE) of ambrosia beetles captured per week across six colored sticky cards (black, blue, green, red, transparent, and yellow) in pecan orchards in Lenox and Waycross, Georgia in field experiment 1. Bars with different lower-case letters indicate significant differences in the number of beetles captured on colored sticky traps (*p* < 0.05, Tukey’s test).

In field experiment 1, the trap color had a significant effect on the capture rates of ambrosia beetles in both locations, Lenox (*χ*^2^ = 149.57, df = 5, *p* < 0.0001) and Waycross (*χ*^2^ = 26.37, df = 5, *p* < 0.0001) (Figure 5, Table S4). In Lenox, red and transparent sticky cards captured significantly higher numbers than all other colored sticky cards except black sticky cards (Figure 5). In Waycross, red sticky cards captured a significantly higher number of ambrosia beetles than black, green, and yellow sticky cards, but not significantly more than transparent and blue sticky cards (Figure 5). In both locations, the yellow sticky card captured significantly fewer ambrosia beetles than all the other colored sticky cards.

**Figure 6:**
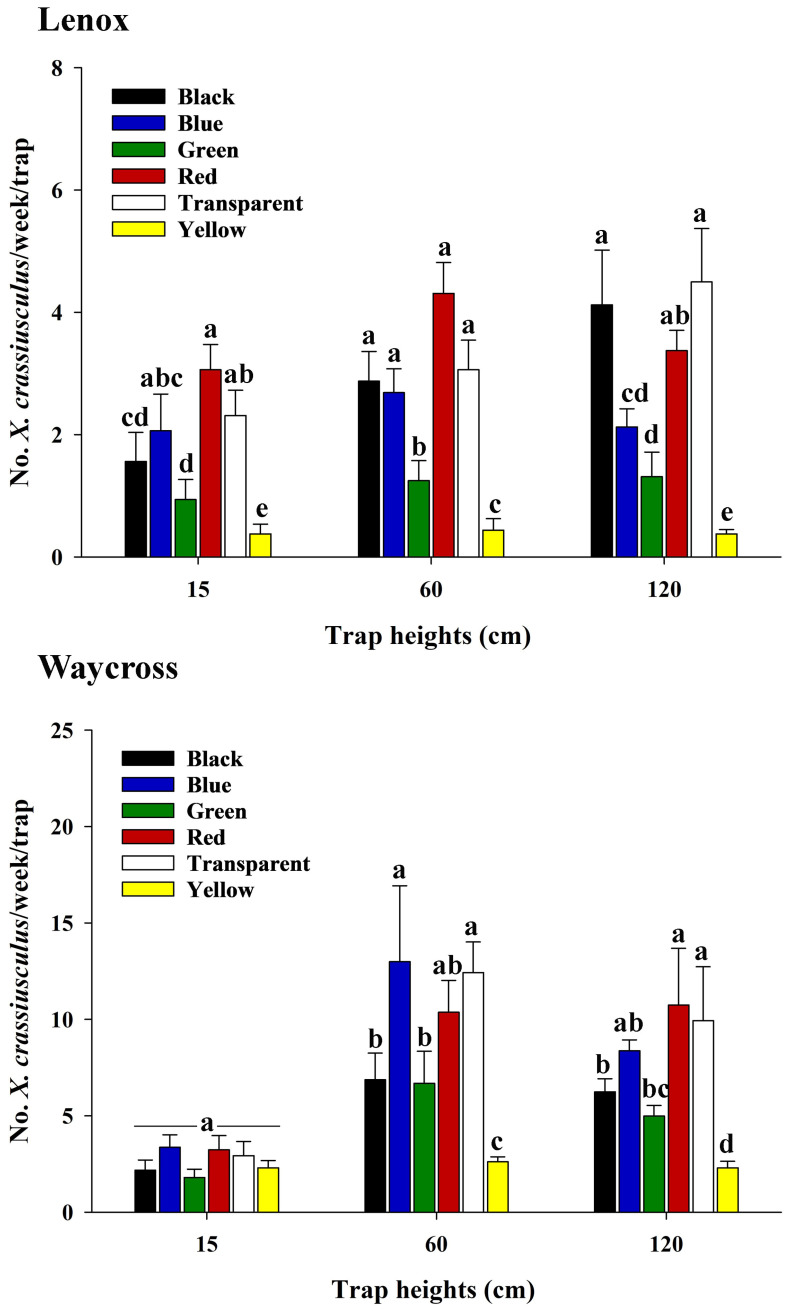
The number (mean ± SE) of *Xylosandrus crassiusculus* captured per week on six colored sticky cards (black, blue, green, red, transparent, and yellow) at 15, 60, and 120 cm from ground level in pecan orchards in Lenox and Waycross, Georgia in field experiment 1. Bars with different lower-case letters indicate significant differences in the number of *X. crassiusculus* captured on colored sticky traps (*p* < 0.05, Tukey’s test).

In both locations, there was a significant interaction between trap color and trap height on the capture of *X. crassiusculus* (Figure 6, Table S4). In Lenox, the red and transparent sticky cards consistently captured more *X. crassiusculus* at all three trap heights than the green and yellow sticky cards (Figure 6). However, there was no significant difference in *X. crassiusculus* captures between blue, red, and transparent sticky cards at 15 cm and 60 cm trap heights. Similarly, there was no significant difference between the captures in black, red, and transparent sticky cards at 60 cm and 120 cm trap heights (Figure 6). In Waycross, blue and transparent sticky cards captured a significantly higher number of *X. crassiusculus* than the black, green, and yellow sticky cards at 60 cm height (Figure 6). However, beetle capture on red sticky cards was statistically equal to the captures on blue and transparent cards. At 120 cm trap height, red and transparent sticky cards captured significantly more *X. crassiusculus* than black, green, and yellow sticky cards, whereas the blue cards were statistically equal to the red and transparent cards. However, at a height of 15 cm from ground level, there was no significant difference in *X. crassiusculus* captures among the colored sticky cards (Figure 6).

**Figure 7:**
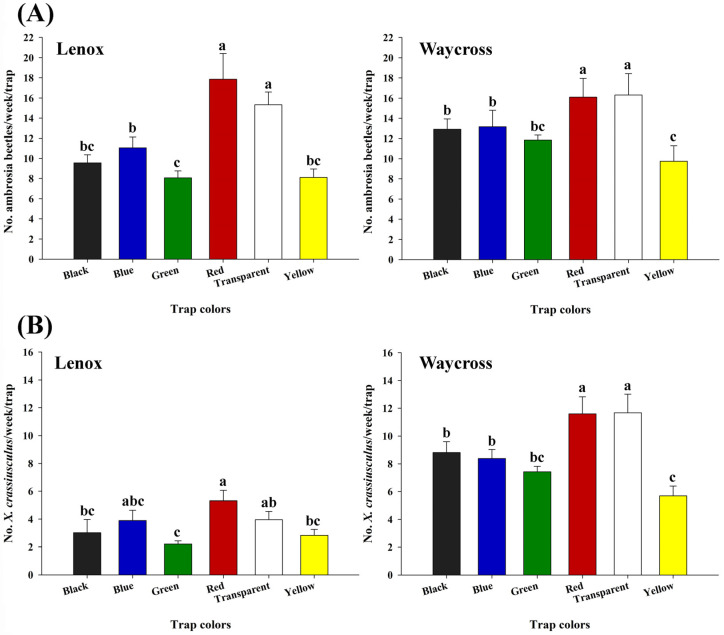
The number (mean ± SE) of ambrosia beetles (**A**) and *Xylosandrus crassiusculus* (**B**) captured per week on six colored sticky cards (black, blue, green, red, transparent, and yellow) in pecan orchards in Lenox and Waycross, Georgia in field experiment 2. Bars with different lower-case letters indicate significant differences in the number of beetles captured on colored sticky traps (*p* < 0.05, Tukey’s test).

Consistent results were observed in experiment 2. The red and transparent sticky cards captured significantly higher numbers of ambrosia beetles in both locations, Lenox (*χ*^2^ = 95.89, df = 5, *p* < 0.0001) and Waycross (*χ*^2^ = 112.50, df = 5, *p* < 0.0001) (Figure 7A). Similar results were observed for *X. crassiusculus* (Figure 7B). In Lenox, red sticky cards captured significantly higher numbers of *X. crassiusculus* than black, green, and yellow sticky cards, but not on transparent and blue sticky cards. However, in Waycross, red and transparent sticky cards captured significantly higher numbers of *X. crassiusculus* compared to all the other colored sticky cards tested (Figure 7B).

### 3.4. Height Preference of Ambrosia Beetles

**Figure 8:**
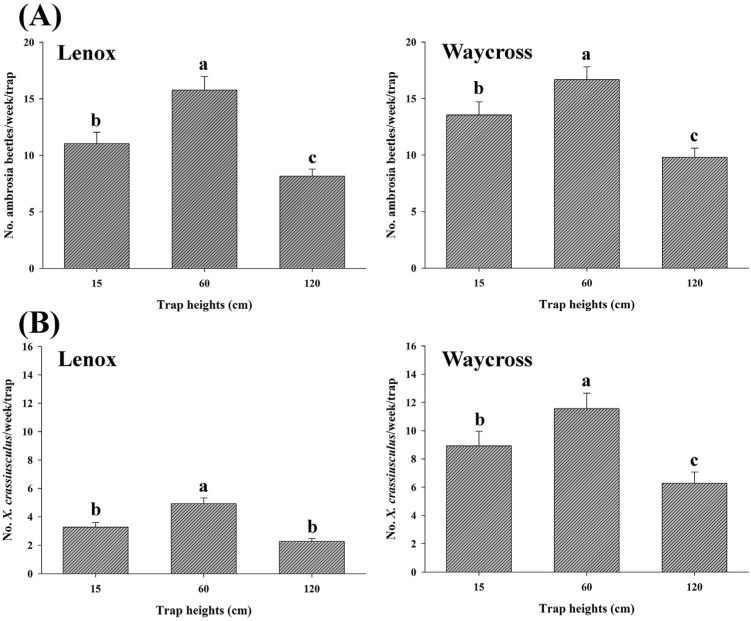
The number (mean ± SE) of ambrosia beetles (**A**) and *Xylosandrus crassiusculus* (**B**) captured per week across three different trap heights (15, 60, and 120 cm from ground level) in pecan orchards in Lenox and Waycross, Georgia in field experiment 2. Bars with different lower-case letters indicate significant differences in the number of beetles captured across the trap heights (*p* < 0.05, Tukey’s test).

In field experiment 2, trap height had a significant effect on the capture rates of ambrosia beetles in both locations, Lenox (*χ*^2^ = 73.26, df = 2, *p* < 0.0001) and Waycross (*χ*^2^ = 165.38, df = 2, *p* < 0.0001) (Figure 8A, Table S5). In both locations, significantly higher numbers of ambrosia beetles were captured at 60 cm compared to 15 and 120 cm from ground level (Figure 8A. Similar results were observed for *X. crassiusculus* for both locations, Lenox (*χ*^2^ = 31.89, df = 2, *p* < 0.0001) and Waycross (*χ*^2^ = 35.23, df = 2, *p* < 0.0001) (Figure 8B, Table S5).

**Figure 9:**
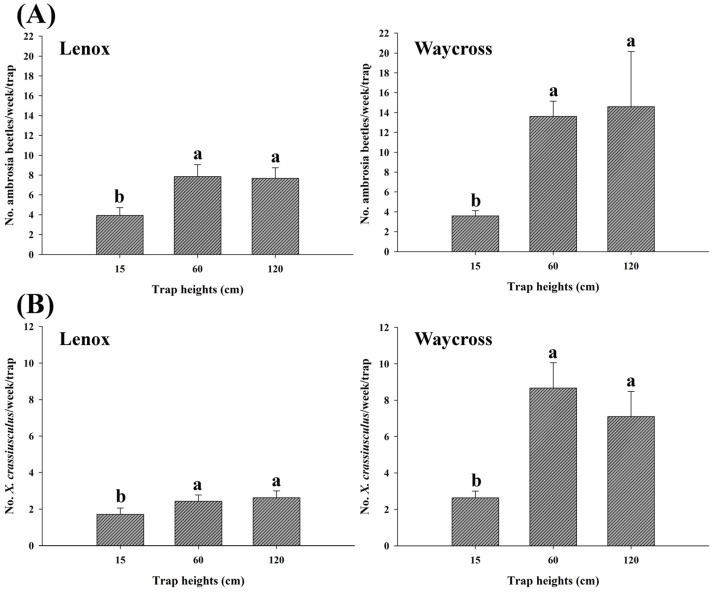
The number (mean ± SE) of ambrosia beetles (**A**) and *Xylosandrus crassiusculus* (**B**) captured per week across three different trap heights (15, 60, and 120 cm from ground level) in pecan orchards in Lenox and Waycross, Georgia in field experiment 1. Bars with different lower-case letters indicate significant differences in the number of beetles captured among the trap heights (*p* < 0.05, Tukey’s test).

The effects of trap height on ambrosia beetle catches in field experiment 1 differed slightly from those in experiment 2. The number of ambrosia beetles captured at 120 cm and 60 cm trap heights were not significantly different from each other. However, the number of ambrosia beetles captured at 60 cm and 120 cm trap heights were significantly higher compared to the beetles captured at 15 cm high traps in experiment 1 in both locations, Lenox (*χ*^2^ = 131.31, df = 2, *p* < 0.0001) and Waycross (*χ*^2^ = 160.23, df = 2, *p* < 0.0001) (Figure 9A). Similar results were also observed for the capture of *X. crassiusculus* (Figure 9B).

### 3.5. Laboratory Based Color Preference of Xylosandrus crassiusculus

**Figure 10:**
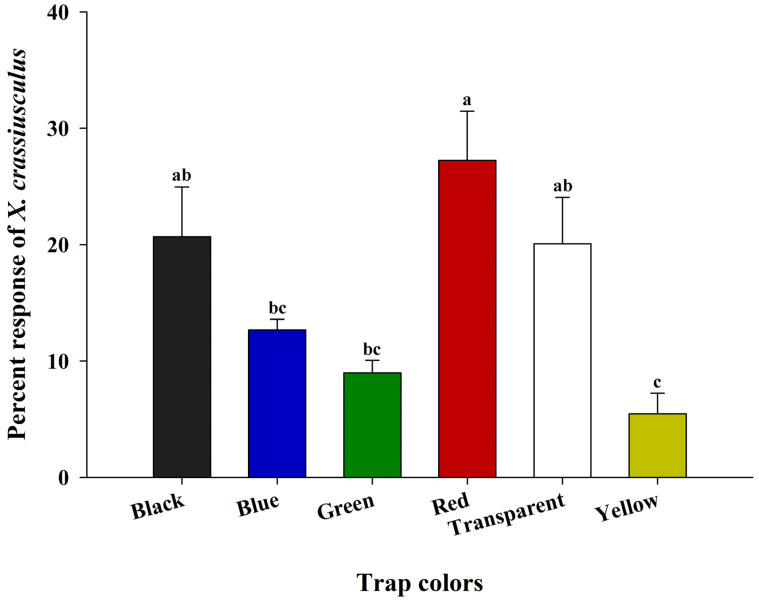
The percent response (mean ± SE) of *Xylosandrus crassiusculus* to six colored sticky cards (black, blue, green, red, transparent, and yellow) in the laboratory setting. Bars with different lower-case letters indicate significant differences in the percent response of *X. crassiusculus* captured on the colored sticky traps (*p* < 0.05, Tukey’s test).

The color of the sticky cards had a significant effect on the preference of *X. crassiusculus* (*F* = 7.084; df = 5, 18; *p* = 0.0008) (Figure 10). Among the six colored sticky cards, significantly higher numbers of *X. crassiusculus* were captured on the red sticky cards compared to the blue, green, and yellow sticky cards, but was not significantly different from the black and transparent sticky cards. Yellow sticky cards captured the lowest numbers of *X. crassiusculus*, and the results were similar to those of the field trial.

## 4. Discussion

The observations made from the natural infestations of ambrosia beetles in pecan trees indicated that three species, *Xylosandrus crassiusculus*, *X. germanus*, and *X. amputatus,* were mainly associated with damage to pecan orchards in Georgia. Among them, *X. crassiusculus* was the most prevalent species in pecan orchards. However, several other ambrosia beetle species, including *X. compactus*, *Xyleborinus saxesenii*, and *Hypothenemus* spp., were recorded from trapping studies in pecan orchards in Georgia [7,21]. These species might be attracted to the ethanol lures used in traps and may come from adjacent woodlots. In the present study (color and height choice experiments) a number of other ambrosia beetle species were recorded, including *X. crassiusculus*, *X. germanus*, *X. compactus*, *X. amputatus*, *Xyleborinus saxesenii*, and *Hypothenemus* spp., and as stated earlier, *X. crassiusculus* was the most dominant species in both pecan orchards.

Various types of traps baited with ethanol lures and ethanol-infused wooden bolts have been proposed to monitor ambrosia beetles [21,43,44]. Tobin et al. [21] suggested that the clear sticky card captured significantly more ambrosia beetles compared to the bottle trap, jar trap, and panel trap. Ambrosia beetles use visual cues for selecting the host and that may influence trap captures among the colored traps [27,45,46]. Previous studies assessed the color preference of ambrosia beetle species, including *X. crassiusculus*, but results were variable, and these studies did not use color sticky traps [24,25,26,27,29,35]. Variation in the capture rate could be due to various reasons, such as trap type, trapping height, ambrosia beetle species composition, and tree habitats. In the current study, the red and transparent sticky cards consistently captured significantly higher numbers of ambrosia beetles in both field experiments and in both experimental locations. Blue and black sticky cards also captured high numbers of ambrosia beetles; however, this trend was not consistent among the experiments and experimental locations. Yellow sticky cards captured consistently fewer ambrosia beetles among all the colored sticky cards in both experiments (field experiments 1 and 2) and in both locations. A similar trend was observed with *X. crassiusculus* in both field and laboratory conditions. Werle et al. [27] reported that more ambrosia beetles were captured in opaque and red colored traps compared to yellow and white traps. Govindaraju and Joseph [24] demonstrated that the purple-colored bottle trap did not capture any additional *X. crassiusculus* or *X. germanus*. In contrast, fewer *X. crassiusculus* and *X. germanus* were captured on the green-colored bottle traps compared to the transparent bottle traps. Similarly, Miller [26] suggested that purple and black-colored multiple-funnel traps captured significantly higher numbers of *X. crassiusculus* than green-colored traps. However, Brar et al. [29] reported that there was no significant influence of trap color (black, white, blue, yellow, red, and transparent) on the capture rate of *Xyleborus glabratus*.

The height of trap placement had a significant effect on the capture of ambrosia beetles [29,30]. The capture rate of *X. glabratus* was significantly greater at trap heights of 35–65 cm and 70–100 cm than 0–30 cm or any height interval above 105 cm [29]. Reding et al. [30] showed that significantly higher numbers of *X. crassiusculus* and *X. germanus* were captured in the trap set 50 cm above the ground compared to the trap set 300 cm above the ground. In this study, we also found evidence of significantly more attacks by ambrosia beetles in the lower portion of the pecan trees (45 cm from ground level) and significantly more beetles were captured in traps placed 60 cm above ground level. However, there was no significant difference in the captures of ambrosia beetles or *X. crassiusculus* captures in traps placed at 60 cm and 120 cm in field experiment 1, where traps at different heights were spaced 20 m apart rather than in the same location (i.e., on the same stake), suggesting that the effect of trap height on ambrosia beetle catch is affected by trap proximity. A similar trend was also observed with *X. crassiusculus*. Thus, it appears that most of the ambrosia beetle species tend to fly close to ground level and their attack is mainly concentrated on the lower portion of the trees.

Along with the ambrosia beetle species, it is also important to understand the effects of the trapping protocol on non-targeted insects, including predators, parasitoids, and pollinators [47]. Therefore, further study should incorporate a more detailed analysis of bycatch to assess the environmental impact and to improve trap specificity.

## 5. Conclusions

Trap color and trapping height are key factors for developing effective trapping protocols for ambrosia beetles. A greater number of ambrosia beetles, including *X. crassiusculus*, were captured using red and transparent sticky cards at a height of 60 cm above ground level. Therefore, utilizing red and transparent sticky cards at this height could enhance trap performance in the ambrosia beetle monitoring program in the pecan production system.

## Data Availability

The original contributions presented in the study are included in the article, further inquiries can be directed to the corresponding author.

## Supplementary Materials

The following supporting information can be downloaded at: https://www.mdpi.com/article/10.3390/insects16060569/s1, Table S1. Sample locations, numbers of infested trees, ambrosia beetle species ratio (%) and distribution of ambrosia beetle entry holes in different height labels of pecan trees. Table S2. Total numbers of beetles captured on various color sticky cards in pecan orchards in Lenox and Waycross, Georgia. Table S3. Total numbers of beetles captured at three trap heights (15 cm, 60 cm, and 120 cm) in pecan orchards in Lenox and Waycross, Georgia. Table S4. Analysis of deviance table (Type II Wald chi-square tests) for the color choice experiment conducted in two pecan orchards, Lenox and Waycross, Georgia. Table S5. Analysis of deviance table (Type II Wald chi-square tests) for height choice experiment conducted in two pecan orchards, Lenox and Waycross, Georgia.
