# Supplementary material for: Effects of Trap Color and Placement Height on the Capture of Ambrosia Beetles in Pecan Orchards
Source: Insects. 2025 May 28;16(6):569. doi: 10.3390/insects16060569 (PMC12192780; doi:10.3390/insects16060569)
Supplement: Supplementary file 1 [file insects-16-00569-s001.zip › insects-3564580-supplementary.pdf]

Supplementary file

Table S1. Sample locations, numbers of infested trees, ambrosia beetle species ratio (%) and distribution of ambrosia beetle entry holes in different height labels of pecan trees

**Locations (County)**	**No. infested tree**	**Ambrosia beetle species (%) (mean ± SE)**			**Average ambrosia beetle entry holes (mean ± SE)**					
		X. cr	***X. ge***	***X. am***	**0-45 cm**	**46-75 cm**	**76-105 cm**	**106-135 cm**	**136-165 cm**	**166-195 cm**
Chula (Irwin)	4	80.5 ± 9.98	14.5 ± 7.66	5 ± 2.53	63.01 ± 4.11	30.94 ± 3.79	5.27 ± 1.86	0.78 ± 0.68	0	0
Portal (Bulloch)	5	93 ± 4.14	7 ± 4.14	0	66.05 ± 4.69	21.91 ± 3.15	6.95 ± 1.39	5.09 ± 1.88	0	0
Quitman (Brooks)	2	100 ± 0	0	0	71.14 ± 20.41	13.41 ± 9.49	7.32 ± 5.17	5.28 ± 3.74	2.84 ± 2.01	0
Waycross (Ware)	1	100 ± 0	0	0	67	19	9	6	0	0

X. cr (*Xylosandrus crassiusculus*), X. ge (*Xylosandrus germanus*), X. am (*Xylosandrus amputatus*)

Table S2. Total numbers of beetles captured on various color sticky cards in pecan orchards in Lenox and Waycross, Georgia

Ambrosia beetle species	Lenox						Waycross					
	Black	Blue	Green	Red	Transparent	Yellow	Black	Blue	Green	Red	Transparent	Yellow
Xylosandrus crassiusculus	132	96	55	158	152	18	279	369	243	356	405	116
X. germanus	23	10	18	24	19	6	32	31	25	25	41	22
X. compactus	17	16	20	34	75	4	37	41	53	48	56	35
X. amputatus	1	1	3	11	8	4	1	3	3	5	0	5
Xyleborinus saxesenii	58	63	40	51	54	42	35	11	19	9	17	25
Hypothenemus sp.	76	48	55	57	69	40	62	66	91	129	45	25
Other	58	68	46	55	38	47	52	32	70	59	34	44

Table S3. Total numbers of beetles captured at three trap heights (15 cm, 60 cm, and 120 cm) in pecan orchards in Lenox and Waycross, Georgia

Ambrosia beetle species	Lenox			Waycross		
	15 cm	60 cm	120 cm	15 cm	60 cm	120 cm
Xylosandrus crassiusculus	314	472	218	858	1111	605
X. germanus	87	118	29	89	67	39
X. compactus	71	167	67	49	83	56
X. amputatus	12	8	17	19	18	14
Xyleborinus saxesenii	160	190	96	98	48	29
Hypothenemus sp.	200	270	184	87	125	97
Other	215	290	174	102	149	102

Table S4. Analysis of deviance table (Type II Wald chi-square tests) for the color choice experiment conducted in two pecan orchards, Lenox and Waycross, Georgia.

Effects	Lenox						Waycross					
	Ambrosia beetles			*Xylosandrus crassiusculus*			Ambrosia beetles			*Xylosandrus crassiusculus*		
	χ^2	Df	p-value	χ^2	Df	p-value	χ^2	Df	p-value	χ^2	Df	p-value
Color	149.57	5	0.0001	162.65	5	0.0001	26.37	5	0.0001	66.67	5	0.0001
Height	131.31	2	0.0001	18.51	2	0.0001	160.22	2	0.0001	116.5	2	0.0001
Color x Height	7.32	10	0.6231	21.56	10	0.0175	12.14	10	0.276	26.23	10	0.0035

Table S5. Analysis of deviance table (Type II Wald chi-square tests) for height choice experiment conducted in two pecan orchards, Lenox and Waycross, Georgia.

Effects	Lenox						Waycross					
	Ambrosia beetles			*Xylosandrus crassiusculus*			Ambrosia beetles			*Xylosandrus crassiusculus*		
	χ^2	Df	p-value	χ^2	Df	p-value	χ^2	Df	p-value	χ^2	Df	p-value
Height	73.26	2	0.0001	31.89	2	0.0001	165.38	2	0.0001	35.23	2	0.0001
Color	95.89	5	0.0001	29.16	5	0.0001	112.5	5	0.0001	35.08	5	0.0001
Height x Color	6.22	10	0.7966	2.22	10	0.9944	13.26	10	0.2095	4.14	10	0.9409
